# Design and optimization of a butterfly-shaped grafting clip and cutting mechanism based on finite element simulation

**DOI:** 10.1371/journal.pone.0339854

**Published:** 2026-01-30

**Authors:** Qiangwen Su, Letian Wu, Qiuxing Yue, Xingdong Gao, Xinwei Cao, Huifeng Shi

**Affiliations:** 1 College of Mechanical and Electrical Engineering, Xinjiang Agricultural University, Urumqi, Xinjiang, China; 2 Agricultural Equipment Research Institute, Xinjiang Academy of Agricultural Sciences, Urumqi, Xinjiang, China; 3 Xinjiang Key Laboratory of Intelligent Control Technology for Facility Agriculture, Urumqi, Xinjiang, China; National University of Sciences and Technology, PAKISTAN

## Abstract

To improve the reliability and cost-efficiency of the grafting mechanism, this study proposes a novel butterfly-shaped polyethylene (PE) grafting clip and integrated cutting-type clip-feeding mechanism. The proposed system replaces traditional steel coil clips and vibration screen feeders with a lightweight, low-damage, and low-cost design capable of continuous clip feeding. The mechanical behavior of the PE clip material was characterized through tensile testing and modeled using the Johnson-Cook constitutive equation. A finite element model of the cutting process was established in Abaqus to investigate the effects of cutting force, speed, and angle on blade stress and clip deformation. Using the Box-Behnken response surface methodology, the cutting parameters were optimized to minimize stress concentration and material distortion. Simulation results showed that optimal performance was achieved at a cutting force of 110 N, a speed of 25.618 cm/s, and a cutting angle of 32.414^°^, yielding a maximum blade stress of 1.31 MPa and clip strain of 5.304%. Validation tests demonstrated that a 30^°^cutting angle produced the highest cutting quality, consistent with simulation predictions. Comparative performance evaluations with a commercial vibrating screen clip feeder confirmed the superiority of the proposed system in terms of reliability, energy efficiency, operational noise, and cost. The developed mechanism offers a compact and practical solution for automated grafting, particularly suitable for small and medium-sized seedling nurseries seeking affordable mechanization technologies.

## Introduction

As the world’s largest vegetable producer and country, China continues to expand the area of facility vegetable cultivation with the growth of market demand [1,2]. However, long-term continuous cropping systems have led to frequent soil-borne diseases and cropping obstacles, becoming a critical bottleneck constraining the industry’s sustainable development [3–5]. Grafting seedling technology is widely recognized as an effective agronomic measure to alleviate these issues due to its significant advantages in enhancing crop disease resistance, stress tolerance, and nutrient use efficiency [6–10]. In recent years, grafting machines, valued for their high operational stability and controllability, are gradually replacing manual grafting and have become a major direction for technological advancement [11,12]. Existing research has primarily focused on aspects such as cutting grafted seedlings, flexible gripping and handling, and image recognition, while studies on clip-feeding mechanisms remain insufficient. As a critical component of grafting machines, the performance of clip-feeding mechanisms directly impacts grafting efficiency and survival rates [13,14]. Therefore, developing a highly reliable and efficient clip-feeding mechanism is crucial for advancing the widespread adoption of automated grafting technology.

Scholars and research institutions at home and abroad have developed a variety of clip-feeding mechanisms [15–19], which can be mainly divided into the following five categories according to the fixing methods of grafted seedlings. First, mechanical clamping fixation applies pressure via external physical fixtures to secure grafted seedlings. Represented by steel coil grafting clip, these are often paired with vibrating screen-type clip-feeding mechanisms. These devices generate significant noise during operation and require frequent replenishment. They suffer from drawbacks such as jamming, damage to grafting clip, and poor machine versatility and stability. Examples include the clip-feeding mechanism developed by Iseki & Co., Ltd. (Japan), the China Hefei JFT-A1500T grafting machine, and the GR300 grafting robot by Atlantic Man (Italy) [20–22]. The second method is needle-based internal fixation, which uses rigid needle-like objects to pierce the rootstock and scion for serial fixation. Ceramic needle fixation is a representative example. The ceramic needles used feature a pentagonal cross-section. This method provides robust fixation and prevents rotational movement at the grafting site. However, it demands extremely consistent stem diameter and seedling age between rootstock and scion, limiting its applicability. Examples include the needle-type grafting machine developed by Korea’s Ideal System [23]. Third is sleeve binding fixation, which employs an elastic sleeve fitted over the graft interface. It relies on the material’s contraction and rebound force to secure the joint, offering advantages such as automatic detachment. However, this method requires custom-made sleeves, resulting in higher costs. Additionally, it is prone to aging in high-temperature environments and mold growth in high-humidity conditions, demonstrating poor environmental adaptability. Due to stringent requirements for stem diameter consistency between rootstock and scion, grafting failure rates are high in practical production. Examples include grafting machines researched by China National Taiwan University, Japan’s Mitsubishi MGM600 model, and Yanmar T600 model [24–27]. Fourth is the wedge grafting method, a “clip-free” fixation technique. The scion is cut into a specific shape. Subsequently, a plug hole is drilled in the rootstock, and the scion is inserted into this drilled hole of the rootstock. The fixation is achieved by friction and mechanical interlock. The machine grafting process imposes stringent requirements on seedling quality consistency, and grafted seedlings carry the risk of mechanical damage. At high operating speeds, grafting misalignment is prone to occur, resulting in a relatively high rework rate. For instance, the 2JC-600 grafting machine developed by South China Agricultural University in China [28]. Fifth is chemical bonding fixation, employing non-mechanical fixation technology. Specific adhesives form a solidified film externally around the graft interface to achieve mechanical fixation without clipping plant stems, causing minimal damage. However, the adhesive is relatively expensive, requires specialized equipment, and has lower operational efficiency. Examples include the GRAFT1000 spray grafting machine by Italy’s TEA Project and the UV adhesive fixation technology developed by Xinjiang Agricultural University [29,30].

Despite the progress made in the aforementioned research, the fundamental contradiction between cost, versatility, and reliability in the clip-feeding mechanism remains unresolved. Existing research has primarily focused on optimizing vibrating screen systems, multi-station outputs, or integrating visual recognition systems. These approaches result in bulky equipment, high costs, and complex structures. Collectively, these factors raise the usage threshold, limiting the adoption and promotion of grafting machines among farmers and seedling enterprises. Consequently, manual grafting remains predominant in China’s grafted seedling production, severely hindering the large-scale application of automated grafting technology.

To address these technical challenges, this paper proposes a method for cutting grafting clip blank wire, designs an automatic clip-feeding mechanism, establishes a 3D model of the cutting component using motion simulation techniques, and constructs a constitutive model for grafting clip blanks through experimentation. Factors influencing clamp cutting effectiveness were analyzed to determine the optimal force, speed, and angle during system operation. The model’s accuracy was validated through cutting trials, culminating in comparative performance tests of clip-feeding mechanisms. The research findings provide technical references for innovative designs of clip-feeding mechanisms in grafting robots.

## Materials and methods

### Selection of grafting clip

**Fig 1:**
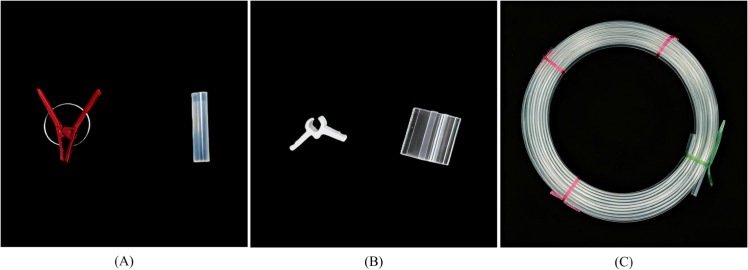
Grafting clip and blank wire. A: Common grafting clip; B: PE Butterfly-Shaped grafting clip; C: Grafting clip blank wire.

Currently, mainstream grafting clip primarily include two types: steel coil spring grafting clip and silicone sleeve grafting clip (Fig 1A). Coil spring grafting clip rely on steel coils to provide clamping force. Their non-adjustable clamping force and self-weight can easily cause mechanical damage to seedlings. Silicone grafting sleeves conform tightly to the cut surface and expand with stem growth. However, they tend to age and harden under extreme conditions such as high or low temperatures. They are suitable for seedlings with thicker stems; otherwise, insufficient fixation strength may result in lower mechanical grafting success rates.

To address these issues, this study designed a transparent polyethylene (PE) butterfly-shaped grafting clip (Fig 1B), which can be cut into individual grafting clip from a grafting clip blank wire (Fig 1C). This material exhibits high chemical stability, environmental non-pollution, and excellent weather resistance, resisting aging, brittleness, or softening. It complies with safety standards for materials in contact with food and pharmaceuticals [31,32]. The grafting clip blank wire is formed into a continuous coil structure via an integrated injection molding and extrusion process. By changing molds, the clip dimensions can be flexibly adjusted to accommodate rootstocks and scions of varying stem thicknesses, significantly enhancing the equipment’s adaptability and operational flexibility.

### Clip-feeding mechanism

The clip-feeding mechanism is the core component ensuring stable and efficient operation of grafting machines. It provides qualified grafting clip for stable and efficient grafting processes, with its reliability directly impacting grafting success rates and overall equipment efficiency. Existing clip-feeding mechanisms lack advantages in spatial efficiency, cost-effectiveness, and stability. These shortcomings hinder the widespread adoption and application of automated grafting technology.

#### Structure and working principle.

**Fig 2:**
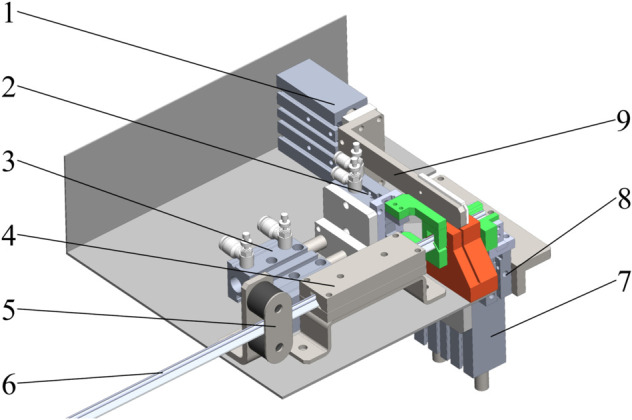
Clip-feeding mechanism. 1: Cutting cylinder; 2: Clamping pneumatic claw; 3: Pushing cylinder; 4: Slide rail; 5: Pulley; 6: Grafting clip blank wire; 7: Lifting cylinder; 8: Feeding pneumatic claw; 9: Cutter holder.

The clip-feeding mechanism is shown in Fig 2, for specific models, see S1 File. A pulley 5 and slide rail 4 are installed upstream of the grafting clip blank wire 6. Adjacent to these, a pushing cylinder 3 is fitted with a clamping pneumatic claw 2 for oriented conveyance and positioning of the grafting clip blank wire. To the right, a cutter holder 9 is connected to a cutting cylinder 1. Downstream of the grafting clip blank wire, a feeding pneumatic claw 8 is mounted on a lifting cylinder 7.

The operational process is as follows: (1) The clamping pneumatic claw holds the grafting clip blank wire. Powered by the pushing cylinder, it moves downstream along the pulley and slide rail; (2) The cutting cylinder pushes the cutter holder forward to sever the grafting clip blank wire, forming a standard butterfly grafting clip; (3) The upstream push cylinder pushes the second time the grafting clip blank wire, insert the grafting clip that have completed the cutting process into the feeding pneumatic claw; (4) The feeding pneumatic claw closes, clips are opened to their fully extended position. The lifting cylinder then moves both the feeding pneumatic claw and the opened clip upward, positioning them for grafting.

#### Design of the cutting component.

**Fig 3:**
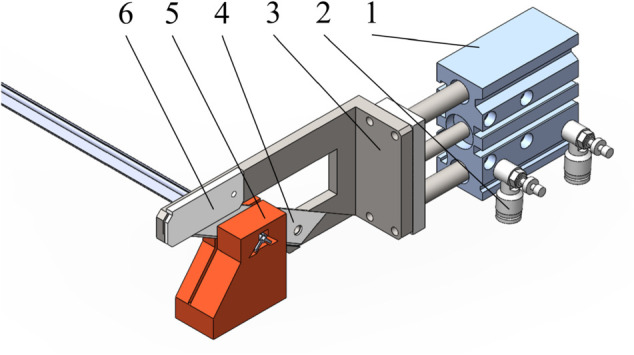
Cutting components. 1: Cutting cylinder; 2: Throttle valve; 3: Cutter holder; 4: Sk5 utility blade; 5: Cutting workbench; 6: Blade guard.

The cutting assembly primarily consists of a cylinder, throttle valve, cutter holder, SK5 utility blade, blade guard, and cutting workbench, as shown in Fig 3. The cylinder is connected to the cutter holder, with the blade securely fastened to the holder via screws. To enhance blade fixation and suppress cutting vibration, an optimally designed blade guard is added to the upper part of the cutter holder. Working in concert with the cutter holder, it ensures the blade maintains stable operation under high-speed, high-frequency impact loads. The cutting workbench used in conjunction with this system features side openings shaped to match the contour of the grafting clip blank wire, allowing the wire to pass through easily. After cutting, the severed clip segment remains within the cutting table. During the next cycle, the leading end of the upstream-fed grafting clip blank wire contacts the severed segment within the table, dislodging it from the cutting table and dropping it into the downstream feeding pneumatic gripper. This design eliminates the need for a separate ejection mechanism, optimizing spatial structure and enhancing motion continuity. This clip-fed wire-cutting method offers significant advantages in improving space utilization and reducing equipment costs, demonstrating strong potential for large-scale application.

#### Selection of power source for cutting components.

To ensure clean cutting of the clip core, the required cutting force must be quantified with sufficient kinetic energy margin. The formulas for calculating the maximum shear force requirement of the selected cutting material and the cylinder thrust are as Eq (1).

$$\left\{ \begin{array}{c} F_{max}=\tau_{max}\times A\\ F=P\times \pi \times {\left(\frac{D}{2}\right)}^{2} \end{array}\right.$$ (1)

In the formula, $F_{\text{max}}$ represents the shear force required to cut the material, in N; $\tau_{\text{max}}$ denotes the material’s shear strength, in N/mm^2^; A is the shear surface area of the material, in mm^2^; F is the cylinder output thrust, in N; P is the cylinder operating pressure, in MPa; D is the cylinder piston rod diameter, in mm. Considering the properties of polyethylene material, its shear strength is set at 5 MPa, and the shear area is calculated to be approximately 20 mm^2^. This yields a theoretical shear force requirement of no less than 100 N. To ensure the timeliness and reliability of the cutting action, a safety factor correction must be applied to the theoretical shear force, taking into account kinetic energy loss, air pressure fluctuations, and safety considerations. Referencing mechanical design standards, a safety factor of 1.2 is applied, resulting in a required maximum output thrust ($F_{\text{max}}$) of approximately 120 N. The cutting cylinder selected is the AirTac TCM16-30 three-axis cylinder. Cylinders produced by this company offer technical advantages within the industry, including stable performance and broad applicability, while also providing cost-effectiveness in equipment cost control [33,34]. According to the manufacturer’s technical specifications, its piston rod radius is 16 mm and thrust stroke is 30 mm, making it suitable for cross-cutting operations on clip-clamped workpieces. At standard operating pressure, it delivers a stable output thrust of 120 N, fully meeting the thrust requirements for clip-clamped workpieces.

#### Design of cutting angle.

The polyethylene (PE) used in grafting clip is a typical soft plastic characterized by low hardness and high toughness. During the cutting process, PE material is prone to significant deformation when subjected to force. Therefore, selecting an appropriate cutting angle is crucial for reducing cutting resistance, suppressing material deformation, and enhancing cutting efficiency.

**Fig 4:**
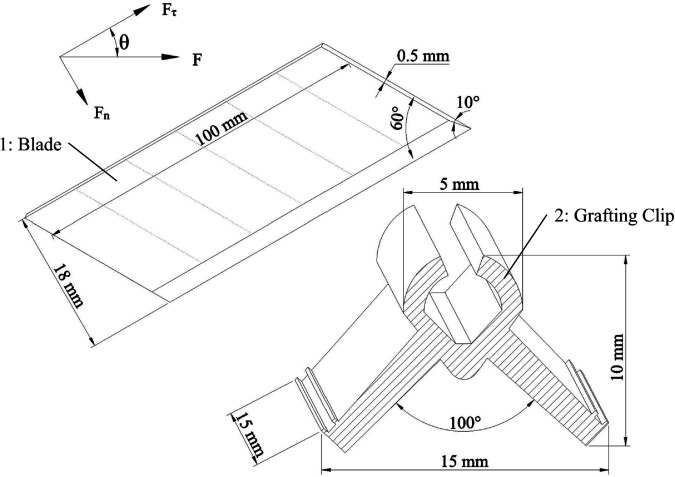
Blade and grafting clip dimensions. 1: Blade; 2: Grafting Clip.

When the blade cuts the grafting clip horizontally to the right,the dimensions of the blade and grafting clip, as shown in Fig 4. Analyzing the forces acting on the blade and clip reveals: A smaller cutting angle *θ* reduces the normal force $F_{\text{n}}$ between the blade and material, lowering friction resistance at the contact surface and enabling effort-saving cutting. Conversely, an excessive normal force intensifies the squeezing effect between the tool and material. This not only increases the contact area and friction resistance, causing substantial plastic deformation in the grafting clip blank wire, but also leads to high stress at the cutting edge, resulting in chipping and dulling.

Analysis indicates that 45^°^ is the critical angle at which the tangential force and normal force reach equilibrium. When the cutting angle is less than 45^°^, the tangential force dominates during the cutting process, generating a sliding cutting effect between the cutting edge and the material surface. This effect reduces cutting resistance while minimizing material damage, thereby significantly enhancing cutting efficiency.

The formulas for calculating the normal force *F*_*n*_, tangential force $F_{\tau }$, and cutting stroke *L* during the cutting process are analyzed as Eq (2).

$$\left\{ \begin{array}{c} F_{n}=F\sin\theta \\ F_{\tau }=F\cos\theta \\ L>\frac{h}{\tan\theta }+\frac{2w}{3} \end{array}\right.$$ (2)

In the formula, *F*_*n*_ represents the normal force, in N; *θ* denotes the cutting angle, in (°); $F_{\tau }$ indicates the tangential force, in N; *L* signifies the cutting stroke, in mm; *h* represents the workpiece clip height, in mm; and *w* denotes the workpiece clip width, in mm.

Considering equipment parameter constraints and the maximum cutting stroke of the equipment cylinder being 30 mm, calculations indicate that the corresponding cutting angle must exceed 24.1°. The optimal cutting angle range is ultimately determined to be 24.1–45°.

#### Determination of factor levels and indicators.

Based on theoretical analysis of the cutting process, the cutting efficiency and surface quality of this cutting component are influenced by a combination of factors including cutting force, cutting speed, and cutting angle. To enhance cutting performance, a simulation analysis of the cutting component’s operational state was conducted. This examined the stress-strain relationship between the blade and the grafting clip blank wire under various parameter conditions, thereby determining the optimal cutting parameters for the component. Cutting force was adjusted by regulating cylinder air pressure, selecting three levels—40 N, 120 N, and 200 N—within the device’s normal operating pressure range. Cutting speed was controlled by adjusting the cylinder throttle valve to regulate action time, setting three speeds: slow (10 cm/s), medium (25 cm/s), and fast (40 cm/s). Cutting angle was tested at three levels—25^°^, 35^°^, and 45^°^—based on preliminary analysis results. This experiment employed the Box-Behnken response surface design methodology to construct a quadratic orthogonal experimental design model with cutting force, cutting speed, and cutting angle as response variables. The experimental factors and level table are shown in Table 1. By varying the three factors across three levels in different combinations, 15 experimental designs were generated, including 3 zero-level tests. These zero-level tests were used to verify the stability of the intermediate levels and for error analysis [35].

**Table 1:** Experimental factors with encoding.

Encoding	Experimental factors		
	Force (N)	Speed (cm/s)	Angle (°)
-1	40	10	25
0	120	25	35
1	200	40	45

### Simulation experiments

#### Constitutive model of materials.

To investigate the performance of cutting components through motion simulation, the Johnson-Cook rate-related plasticity method was employed to establish a constitutive model for the clip material, describing its mechanical behavior [36]. This model offers the advantages of a concise form and parameters that can be conveniently calibrated through tensile tests. It effectively characterizes the strain rate sensitivity of materials and adapts to the simulation requirements of plastic flow, plastic deformation, and strain hardening processes at different strain rates. It provides effective support for in-depth analysis of cutting simulations. Relevant plastic deformation expressions are as Eq (3). It should be noted that this simplified model has certain limitations: First, it does not account for localized temperature increases that may occur during the cutting process, nor the resulting thermal softening and temperature-dependent evolution of material properties. Second, it fails to adequately capture the inherent viscoelastic and time-dependent characteristics of PE materials. Additionally, it disregards the potential effects of material anisotropy and ambient temperature variations on performance.

$$\sigma =\left(A+B\varepsilon^{n}\right)\left(1+C\ln\frac{\dot{\varepsilon }}{{\dot{\varepsilon }}_{0}}\right)\left[1-{\left(\frac{T-T_{r}}{T_{m}-T_{r}}\right)}^{m}\right]$$ (3)

In the equation, *σ* represents the equivalent flow stress, in MPa; *ε* denotes the equivalent plastic strain; $\dot{\varepsilon }$ is the current strain rate, in s^−1^; ${\dot{\varepsilon }}_{0}$ is the reference strain rate, s^−1^; *T* is the current temperature, in °C; *T*_*r*_ is the reference temperature (typically room temperature), in °C; *T*_*m*_ is the melting point temperature of the material, in °C; *A* denotes the initial yield strength of the material at the reference temperature and reference strain rate, in MPa; *B* and *n* jointly describe the strain hardening behavior of the material, characterizing the change in strength with increasing plastic strain; *C* is the strain rate strengthening coefficient, reflecting the effect of increased material strength with rising strain rate; *m* is the thermal softening index, used to quantify the decrease in material strength due to temperature increase.

#### Tensile test.

**Fig 5:**
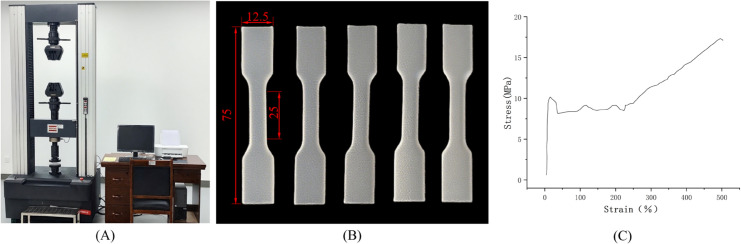
Experimental materials and data. A: Electronic universal testing machine; B: Tensile specimen; C: Stress-Strain curve.

Using an electronic universal testing machine (Fig 5A), axial tensile tests were conducted on polyethylene specimens (Fig 5B). Mechanical properties, including elastic modulus, Poisson’s ratio, and yield strength, were estimated based on relevant formulas [37]. The measured experimental data (S2 Table) were fitted using MATLAB to generate stress-strain curves, yielding the experimental results for tensile failure of the grafting clip blank wire (Fig 5C). The material exhibited a distinct yield point and strain softening phenomenon, followed by a necking stage and a strain hardening stage. Analysis of the images determined the elastic modulus E of the grafting clip blank wire to be 207 MPa, with a tensile strength limit between 10 and 11 MPa. The corresponding Johnson-Cook model parameters are shown in Table 2.

**Table 2:** Parameters of the material model.

*A* (MPa)	*B* (MPa)	*n*	*m*	*C*
10.1	2.5	0.3	1	0.029

#### Finite element model.

**Fig 6:**
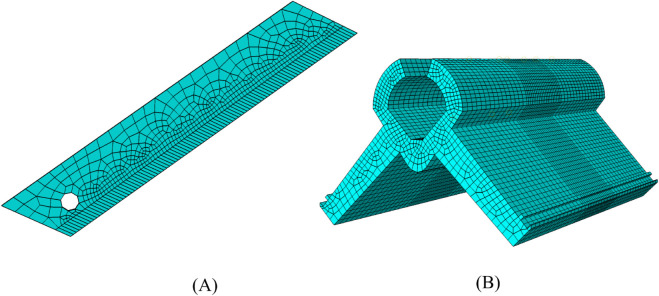
Finite element model. A: Blade mesh partitioning; B: Grafting clip mesh partitioning.

To enhance simulation efficiency and simplify the simulation model of the cut component, the blade and the grafting clip were selected for simulation. First, three-dimensional models of the blade and grafting clip were created in SolidWorks. These were then imported into Abaqus software for simulation (S3 Video), which offers significant advantages in handling nonlinear deformation of plastic materials. A simulation model was constructed based on explicit dynamics, incorporating material parameters such as elastic modulus and Poisson’s ratio into the model via the property module (Table 3). To address the high stress-strain gradients in the cutting region, the mesh in the cutting area was locally refined to enhance solution accuracy. Both the blade (Fig 6A) and the grafting clip (Fig 6B) were meshed using C3D8R linear reduced-integration hexahedral elements. This element type ensures computational accuracy while offering good numerical stability and computational efficiency. To simulate the interaction between the cutting component and the clamping structure, surface-to-surface contact is defined at the interface of the blade and the clip. For tangential contact, the penalty method is adopted with a friction coefficient of μ=0.26 . For normal contact, a hard contact algorithm is used to allow separation after contact, which conforms to the actual mechanical behavior of the structure during cutting. A fixed constraint is imposed on the base surface of the clip to restrict its rigid body motion, while the blade is treated as a rigid body and a displacement constraint is applied to realize the cutting movement. Considering computational efficiency and simulation accuracy, the total active time of the explicit dynamic simulation is set to 0.01 s, which is sufficient to capture the key plastic deformation and contact response of the material during the cutting process.

**Table 3:** Physical properties of blade and grafting clip.

Parameters	Density ρ	Young’s modulus *E*	Poisson’s ratio ν
SK5 Blade	0.921g/cm^3^	207MPa	0.28
PE Grafting Clip	7.83g/cm^3^	205000MPa	0.44

#### Grid sensitivity and convergence tests.

Finite element problems involving contact mechanics often exhibit high stress-strain gradients at the cutting edge. To evaluate numerical errors and ensure the credibility of simulation results, mesh sensitivity analysis is therefore necessary. Four progressively refined meshes (denoted as M1 to M4) with different densities were established in the contact refinement region, where the element sizes on both sides of the contact surface were controlled to a ratio of ≤ 2:1 to minimize contact projection errors. For each mesh, two key indicators were recorded: the peak von Mises stress (MPa) at the tool tip and the peak equivalent plastic strain (%) in the fixture contact zone. The variations of these indicators with element size were analyzed, and the relative changes between adjacent mesh refinements were calculated. The detailed results are presented in Table 4.

**Table 4:** Mesh convergence results.

Mesh ID	Size (mm)	No. of Mesh	Stress (MPa)	Strain (%)	Relative Change (%)
M1	0.4	14,552	1.46	6.05	—
M2	0.3	20,812	1.34	5.45	stress: −8.2%; strain: −9.9%
M3	0.2	29,862	1.31	5.30	stress: −2.24%; strain: −2.75%
M4	0.1	53,148	1.29	5.27	stress: −1.53%; strain: −0.57%

Table presents the mesh simulation convergence results. It can be observed that the relative change in key indicators (peak stress and strain) from Mesh M2 to M3 decreases to less than 3%, indicating that Mesh M3 (with a mesh size of 0.2 mm) has essentially met the convergence criteria. The relative change further diminishes when refining from Mesh M3 to M4, which verifies the numerical stability of the simulation results.

## Results and discussion

### Simulation results

**Fig 7:**
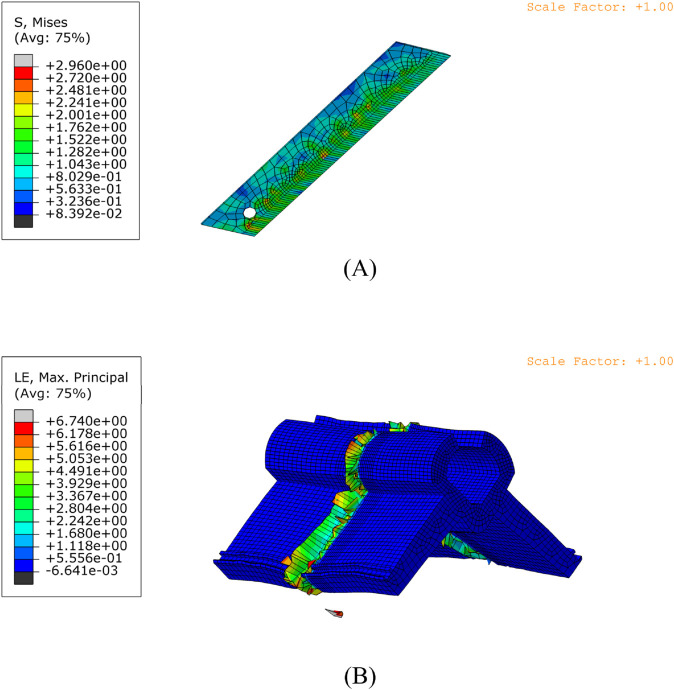
Cutting simulation. A: Blade stress; B: Clip strain.

Through simulation calculations (Fig 7), the results of the Mises stress distribution of the cutting tool and the true strain (LE) of the grafting clip under each set of parameter test conditions were recorded. The obtained results are shown in Table 5.

**Table 5:** Experimental protocol and results.

Serial Number	Experimental factors			Blade Stress	Clip Strain
	Cutting Force *A*	Cutting Speed *B*	Cutting Angle *C*	*Y* (MPa)	*Z* (%)
1	-1	-1	0	1.96	6.24
2	1	-1	0	2.96	6.74
3	-1	1	0	1.83	6.36
4	1	1	0	2.83	6.78
5	-1	0	-1	2.45	6.12
6	1	0	-1	2.45	6.68
7	-1	0	1	2.93	6.86
8	1	0	1	4.41	7.16
9	0	-1	-1	2.15	5.68
10	0	1	-1	2.71	5.83
11	0	-1	1	3.97	6.53
12	0	1	1	3.77	6.76
13	0	0	0	1.40	5.39
14	0	0	0	1.40	5.39
15	0	0	0	1.40	5.39

### Analysis of test results

Simulation results reveal the effects of cutting force, cutting speed, and cutting angle on blade stress and clamp strain during material cutting operations, with all values being relatively small. These values were imported into Design-Expert software to analyze the experimental results of blade cutting grafting clip using analysis of variance (ANOVA). The ANOVA and significance test results for the regression model are shown in Table 6, for detailed data see (S4 File). A quadratic polynomial regression model was established. The regression equations for blade cutting stress Eq (4) and actual strain of the grafting clip Eq (5) are as follows.

$$Y=\;1.40+0.435A+0.0125B+0.6650C+0AB+0.37AC\;\;-0.19BC+0.4525A^{2}+0.5425B^{2}+1.21C^{2}$$ (4)

$$Z=5.39+0.2225A+0.675B+0.375C-0.02AB-0.065AC\;+0.02BC+0.8225A^{2}+0.3175B^{2}+0.4925C^{2}$$ (5)

**Table 6:** Experimental protocol and results.

Source	Blade stress Y				Clip Strain Z			
	Sum of Squares	df	F	P	Sum of Squares	df	F	P
Model	12.28	9	64.33	0.0001	4.95	9	60.13	0.0001
A	1.51	1	71.37	0.0004	0.396	1	43.33	0.0012
B	0.0013	1	0.0589	0.8178	0.0364	1	3.99	0.1023
C	3.54	1	166.8	<	1.13	1	123.09	0.0001
AB	0	1	0	1	0.0016	1	0.1751	0.693
AC	0.5476	1	25.82	0.0038	0.0169	1	1.85	0.232
BC	0.1444	1	6.81	0.0477	0.0016	1	0.1751	0.693
A^2^	0.756	1	35.64	0.0019	2.5	1	273.29	< 0.0001
B^2^	1.09	1	51.23	0.0008	0.3722	1	40.72	0.0014
C^2^	5.38	1	253.82	<	0.8956	1	97.99	0.0002
Residual	0.1061	5			0.0457	5		
Lack of fit	0.1061	3			0.0457	3		
Errors	0	2			0	2		
Total	12.39	14			4.99	14		
	*R*^2^ = 0.9914, Adj *R*^2^ = 0.9760				*R*^2^ = 0.9908, Adj *R*^2^ = 0.9744			

Table 5 shows that the model validation results indicate *R*^2^ values of 0.9914 and 0.9908, respectively, with adjusted *R*^2^ values of 0.9760 and 0.9744. This demonstrates that the model exhibits a high degree of fit with the experimental data and effectively reflects the relationship between parameters and response values. The p-values for both the blade stress model and the clip strain model are highly significant (*p* < 0.01), making them suitable for experimental testing and analysis. The variance analysis for blade stress revealed that the cutting force A, cutting angle C, and interaction term AC were less than 0.01, indicating these factors exerted extremely significant influence on the magnitude of blade stress. The interaction term *BC* < 0.05, signifying this factor significantly affected blade stress magnitude. Cutting speed B and interaction term *AC* > 0.05 indicated these factors had no significant impact on blade stress magnitude. The variance analysis of the clip strain revealed that the cutting force A and cutting angle C were less than 0.01, indicating these factors exerted an extremely significant influence on the magnitude of blade stress. The cutting speed B and interaction terms AB, AC, and BC were greater than 0.05, suggesting these factors did not significantly affect the magnitude of blade stress. Furthermore, non-significant regression terms were removed to ensure the significance of the regression equation model. By re-fitting blade stress and fixture strain using regression equations, we obtained Eqs (6) and (7).

$$Y=1.40+0.435A+0.6650C+0.37AC\;\;-0.19BC+0.4525A^{2}+0.5425B^{2}+1.21C^{2}$$ (6)

$$Z=5.39+0.2225A+0.375C+0.8225A^{2}+0.3175B^{2}+0.4925C^{2}$$ (7)

**Fig 8:**
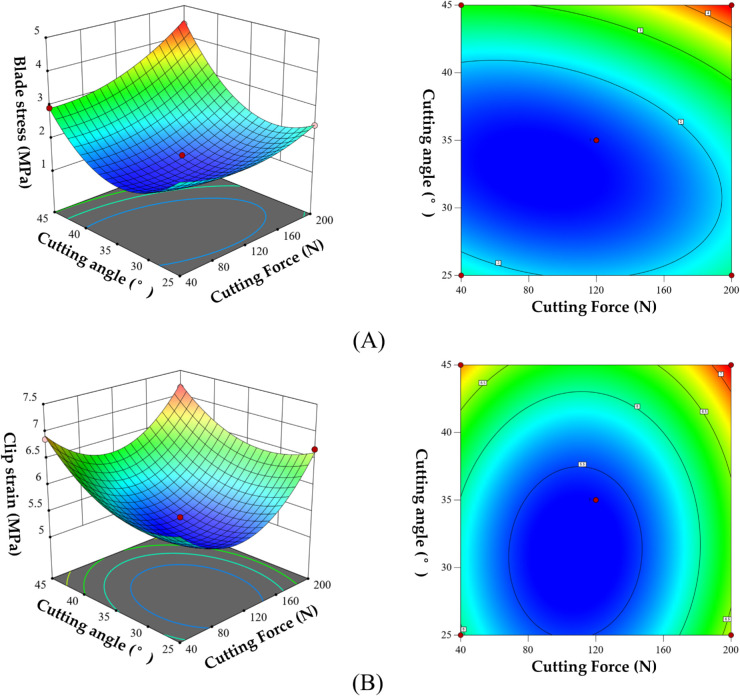
Response surface analysis. A: Effect of the interaction between cutting angle and cutting speed on blade stress; B: Effect of the interaction between cutting angle and cutting speed on clip strain.

By analyzing the regression coefficients above, it can be observed that the primary and secondary factors influencing blade stress and clip strain differ. The order of influence for blade stress is: applied force A, cutting angle C, speed B. For clip strain, the order is: cutting angle C, applied force A, speed B. Furthermore, the interactions among applied force, speed, and cutting angle are analyzed, as shown in Fig 8.

As shown in Fig 8, When the cutting speed and cutting force remain constant, the blade stress initially decreases and then increases with the increase of the cutting angle, while the clip strain shows a clear upward trend. This occurs because, in the initial stage of increasing the cutting angle, the contact mode between the blade and the clip blank approaches an ideal shear state, resulting in reduced frictional resistance and extrusion components, which lowers the blade stress and keeps the clip strain relatively small. However, as the cutting angle continues to increase, the interaction between the blade and the material shifts from being shear-dominated to extrusion-dominated. The normal force per unit area at the blade tip increases significantly, intensifying the local stress concentration effect, which in turn leads to a gradual increase in blade stress and a notable increase in clip deformation.

When the cutting speed and cutting angle are held constant, the blade stress increases with the rise in cutting force, whereas the clip strain first decreases and then increases. This can be attributed to the stable contact state between the tool and the clip blank, where stress is approximately proportional to the applied force—meaning that a greater force results in higher stress, thus causing the blade stress to gradually increase with cutting force. As for the clip strain, it slightly decreases with increasing force at lower levels, reflecting a degree of structural adaptability. However, as the cutting force continues to rise, the clip undergoes significant plastic deformation under high load, leading to a marked increase in strain.

The above results indicate that the cutting force, cutting speed, cutting angle, and their interactions significantly influence blade stress. To obtain optimal matching parameters for blade stress and clip strain, multi-objective optimization must be performed with dual goals of “minimizing blade stress” and “minimizing clip strain” (balancing the need to reduce blade stress concentration and minimize excessive clip plastic deformation). For this purpose, a parametric mathematical model is established to solve for optimal parameters through objective functions and constraints, as Eq (8).

$$\left\{ \begin{array}{c} \min Y\\ \min Z\\ 40\;\text{N}⩽A⩽200\;\text{N}\\ 10\;\text{cm/s}⩽B⩽40\;\text{cm/s}\\ 25^{\circ }⩽C⩽45^{\circ } \end{array}\right.$$ (8)

Using the Design-Expert software’s results optimization module to solve for the target, the following optimal parameters were obtained: At a cutting force of 110 N, cutting speed of 25.618 cm/s, and cutting angle of 32.414^°^, the blade stress and clip strain index for cutting grafted clip blank wire are minimized under these conditions, specifically 1.31 MPa and 5.304%, respectively. This configuration enables efficient cutting and feeding of the clips.

### Confirmatory testing

Based on the optimization results, a prototype clip-feeding mechanism was successfully developed at the Agricultural Equipment Research Institute of the Xinjiang Academy of Agricultural Sciences. By adjusting the pneumatic pressure valve, the cylinder force was stabilized at 110 N. Utilizing a throttle valve, the cutting speed was controlled at 25 cm/s. Cutting tests were conducted with blade angles fixed at 20^°^, 30^°^, and 40^°^ using a blade guard. The system evaluated the cut quality and deformation behavior of grafting clip during the cutting process. The cutting performance evaluation centered on whether the cut surface morphology met process specifications, focusing on detecting defects such as burrs, tears, indentations, irregular protrusions, and plastic deformation. Each cutting angle group underwent 100 repeated effective cuts, with the cutting stroke and grafting clip pass rate (Y) statistically analyzed. The formula is defined as Eq (9).

$$Y=\left(\frac{X_{1}}{X}\right)\times 100\%$$ (9)

**Fig 9:**
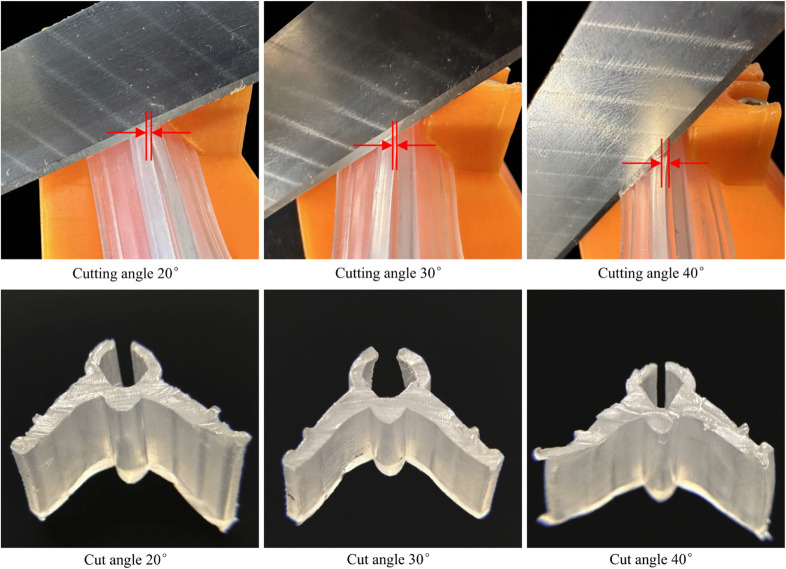
Degree of deformation in grafting clip and cutting effect at different cutting angles.

In the formula, X1 represents the number of qualified, defect-free graft clips; X denotes the number of graft clips subjected to the cutting test. To precisely capture the cutting dynamics, a high-speed camera system was employed to record the cutting process at a high frame rate, enabling measurement of deformation and analysis of failure modes. Photographs of the experimental process and statistical results are shown in Fig 9 and Table 7, for detailed data see (S5 Table).

**Table 7:** Parameters at different cutting angles.

Cutting angle (°)	Def. Length (mm)	Cut. stroke of blade (mm)	Grafting Clip Qual. Rate (%)
20	0.29±0.11	37.47	67
30	0.36±0.14	27.32	88
40	0.43±0.19	21.92	56

The test employed a cutting direction from left to right. Results indicate that the cutting angle significantly impacts notch quality: at a 20^°^ angle, the left side of the notch appears smoother, but burrs form at the right-hand tail end. This occurred because the smaller angle increased the contact area between the blade and the clip, raising cutting resistance and causing material adhesion, which formed burrs at the right exit. At a cutting angle of 30^°^, the cut surface was uniformly smooth and flat, yielding the most ideal results, indicating the high reliability of the optimization model. When the cutting angle increased to 40^°^, significant plastic deformation occurred on the left side of the cut, while the right side became relatively rough. This occurs because as the angle increases, the normal force significantly rises while the skiving effect weakens. Consequently, the tool separates the material by compression, not only accelerating edge wear but also causing severe plastic deformation of the clips. This study revealed that the cutting angle significantly impacts cut quality, identifying 30^°^ as the more favorable angle. This angle balances normal force and shear action, simultaneously achieving high-quality cuts while reducing tool wear. This discovery provides critical theoretical foundations and practical guidance for optimizing cutting process parameters in precision manufacturing.

### Cross-comparative testing

**Fig 10:**
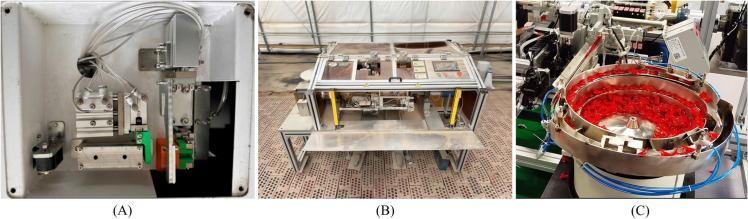
Clip-feeding mechanism and grafting machine. A: Prototype of cutting-type clip-feeding mechanism; B: Jiafute JFT-A1400T semi-automatic grafting machine; C: Vibrating screen clip-feeding mechanism.

To systematically evaluate the technical feasibility and overall performance of the independently developed cutting-type clip-feeding mechanism (Fig 10A), this study conducted comparative tests using the commercially available JFT-A1500T semi-automatic grafting equipment (Fig 10B) as a reference. This equipment employs a vibrating screen-type clip-feeding mechanism (Fig 10C), is widely used in small-to-medium-sized nurseries, and possesses market representativeness, making it suitable as a performance benchmark.

Environmental variables were strictly controlled during testing to ensure both types of clipping devices operated under equivalent conditions. The test cycle was set to one hour of continuous operation, with relevant data collected synchronously throughout the device’s operation. The statistical results as shown in Table 8.

**Table 8:** Clip-feeding mechanism comparison chart.

Clipping Method	Clip-cutting	Vibrating screen
Dimensions (mm)	260 × 240 × 90	500 × 500 × 600
Drive Method	Pneumatic	Electric motor
Supply Method	Pneumatic Claw	Friction vibration
Noise Level	Lower	Higher
Energy Consumption	Lower	Higher
clipping Speed (pieces/hour)	Approximately 2000	Approximately 1800
Number of failures	0	2
Equipment Cost Price (RMB)	3000	>8000

Analysis of the above table reveals that the vibration screen clip-feeding mechanism exhibits multifaceted structural and functional shortcoming. Operating at excessively high speeds can easily cause clip jamming and blockages, thereby reducing reliability. Continuous operation of the vibrating motor not only generates high noise levels, leading to operator fatigue, but also results in persistently high energy consumption. Furthermore, the device is highly sensitive to environmental conditions and material states. For instance, burrs on grafting clip edges, variations in track friction coefficients due to windy and sandy conditions in Xinjiang of China, or excessive clip filling in the hopper can all cause feeding interruptions, necessitating frequent manual intervention and severely limiting overall production efficiency. The mechanical structure of the vibrating screen also requires high maintenance costs, with parameter adjustments requiring specialized technicians, further increasing operational complexity.

In contrast, the cutting type clip-feeding mechanism demonstrates significant advantages in several key performance aspects. Its compact design facilitates seamless integration and layout within automated grafting systems. Operating at low noise levels, it substantially improves workplace comfort, making it suitable for high-speed automated production environments while accommodating diverse clip conditions and complex operational scenarios. Employing an intermittent work mode with on-demand operation enables more efficient energy consumption control. The feeding process is streamlined, requiring no mid-cycle replenishment after initial clipping, achieving high automation. Crucially, its mechanical design fundamentally eliminates common vibration screen issues like clogging and jamming, ensuring continuous clip feeding and operational stability. In summary, the cutting-type clip feeding device excels in efficiency, reliability, environmental adaptability, and cost-effectiveness. It provides a high-performance, low-cost advanced solution for automated grafting systems, with overall performance significantly surpassing traditional vibrating screen clip-feeding method [38].

Through comparative analysis, this study demonstrates that the designed cut-and-clamp grafting clip significantly reduce costs compared to traditional steel-coil clamps while eliminating damage to seedlings during clamping. It confirms that the cut-type clip-feeding mechanism fundamentally resolves the reliability, environmental adaptability, and economic deficiencies inherent in traditional vibrating screen systems. Combined with its complementary low-cost, highly adaptable transparent PE grafting clip blank wire, this system collectively provides a high-performance, highly stable solution for automated grafting.

### Development of grafting machines

**Fig 11:**
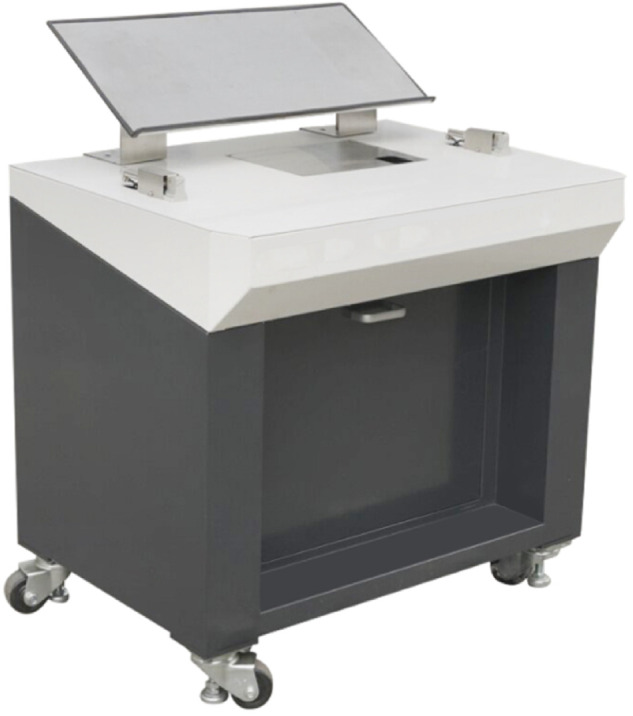
Prototype of the clip-type grafting machine.

Based on prior structural design and mechanism research of key components for grafting machines, the joint team at the Agricultural Equipment Research Institute of the Xinjiang Academy of Agricultural Sciences has successfully developed a lightweight, simplified cutting-clip grafting machine, completing its prototype fabrication and assembly (Fig 11). This equipment is suitable for grafting seedlings of eggplant, tomato, pepper, and certain melon varieties. The new device integrates key functions such as separate angled cutting of scions and rootstocks and an automatic clip-feeding mechanism. All components underwent rigorous performance testing to ensure grafting success rates and production efficiency. Calculations show its production cost is only 10,000 yuan. Compared to market alternatives like vibrating screen-type or robotic arm-type grafting machines costing over 300,000 yuan, it offers significant cost advantages. This effectively reduces the initial equipment investment burden for small and medium-sized seedling nurseries and seedling-producing farmers.

To validate the equipment’s performance, the trial selected the locally representative tomato cultivar “Tomato Nova 101” as the scion and “Tolubam” as rootstock (both with good compatibility). Healthy seedlings with stem diameters of 3.5±0.2mm and at the three-true-leaf stage were selected as experimental materials. Three treatment groups were established: manual grafting, commercial machine grafting (JFT-A1200T semi-automatic grafting machine with vibrating screen clip feeder), and a sample grafting machine group. Each group completed grafting on 532 rootstocks and scions respectively. The manual group was operated by one skilled worker following standard procedures. The commercial machine group involved two operators placing rootstocks and scions according to protocol after equipment calibration. The sample machine group was operated by one trained personnel using the device. Grafting efficiency was calculated by recording completion times. A graft was considered successful if the grafting clip transitioned smoothly from the clamp delivery system to the grafting seedling. grafting clip were deemed unsuccessful if clip jamming, clip drop, or operational disruption occurred. Detailed records were maintained, and grafting success rate Eq (10) and 14-day graft survival rate Eq (11) were statistically analyzed.

$$Y_{1}=\left(\frac{Z_{1}}{Z}\right)\times 100\%$$ (10)

$$Y_{2}=\left(\frac{Z_{2}}{Z_{1}}\right)\times 100\%$$ (11)

In the formula, *Z*_1_ represents the number of grafted seedlings successfully completed; *Z* denotes the number of rootstock (scion) seedlings used for grafting operations; *Z*_2_ indicates the number of grafted seedlings that survived. A comprehensive comparative analysis was conducted based on grafting speed, success rate, survival rate, and production costs, with the recorded results shown in Table 9.

**Table 9:** Comparative analysis of prototype machines, manual, and commercial devices.

Source	Artificial grafting	JFT-A1200T Machine	Sample Machine
Number of operators	1 person	2 person	1 person
Clipping Method	Self-pickup	Vibrating Screen	Clip-cutting
Grafting Speed (plants/hour)	Approximately 310	Approximately 1500	Approximately 700
Grafting Success Rate (%)	99.8	97.4	98.4
Graft Survival Rate at 14 Days (%)	88.5	95.7	96.8
Price (RMB)	≥200yuan per day	300000yuan per unit	20000Yuan per unit

Test results indicate that in terms of grafting efficiency, manual grafting achieves approximately 300 plants per hour with a noticeable decline over time. Commercially available units reach about 1,500 plants per hour but experienced three instances of vibration screen jamming. The sample grafting machine operates at approximately 700 plants per hour with continuous, stable, and trouble-free operation. Performance metrics reveal the prototype grafting machine achieved a 98.4% grafting success rate and a 96.8% 14-day survival rate. Its operational efficiency is 2.3 times that of manual grafting, saving labor costs by 800 yuan per hectare. Its clip-and-cut feeding device reduces environmental noise and minimizes structural space requirements. While ensuring high cost-effectiveness, it resolves the issue of unstable survival rates caused by technical variations in manual grafting. Additionally, the compact design facilitates portability and adapts to diverse operational environments. Its affordable price point promotes the mechanization of grafting techniques. However, the use of existing off-the-shelf components and modular solutions for its internal structure limits further optimization potential. The compact, low-cost, and highly stable clip-grafting machine developed in this study successfully achieves a unified balance of high-efficiency operation (700 plants per hour), high survival rate (96.8%), and favorable economic viability. It provides a more reliable and easily scalable practical solution for mechanized grafting.

## Conclusion

This study effectively tackles the prevailing challenges in current grafting machine feeding mechanisms, including low operational efficiency, excessive structural bulk, and high production costs. By introducing a novel cutting-based feeding approach, this study develops polyethylene (PE) butterfly-shaped grafting chips derived from blank wire research, which achieves fundamental innovation. This substitution of conventional steel coil clamps with lightweight, customizable PE clips significantly enhances the feasibility and cost-effectiveness of automated grafting.

Through mechanical testing of the grafting clips, the material parameters for simulation were calibrated using the Johnson–Cook model, whose concise form and easily obtainable tensile-test parameters allow effective representation of strain-rate–dependent plastic deformation during cutting. Based on the simulated blade stress and clip strain responses, the internal relationship between cutting mechanics and incision quality was clarified, and response-surface analysis yielded optimal cutting parameters of 110 N, 25.618 cm/s, and 32.414^°^, ensuring stable and high-quality cutting performance. It should be noted that the Johnson–Cook model is a simplified description that does not consider local thermal effects, viscoelasticity, or potential anisotropy of PE; these aspects constitute important directions for future research to further enhance the realism and accuracy of cutting simulations.

Comparative validation involving different cutting angles further confirmed the predictive reliability and accuracy of the finite element configurations. Test results demonstrated that the $30^{\circ }$ cutting angle produced favorable results, closely matching the simulated outcomes. This alignment underscores the practical value of the optimization model in mechanical design and parameter selection, and demonstrates the significant potential of finite element simulation as a time-saving R&D methodology. By leveraging simulation, the development cycle for key components was significantly shortened, experimental costs were reduced, and a robust theoretical foundation was established for improving the operational performance of the clip-feeding mechanism and the overall system.

To address the issues of bulky size, high cost, and limited adoption among ordinary farmers in existing grafting equipment, this study successfully prototyped a compact, low-cost, cut-and-clamp grafting machine. Comparative trials demonstrate the device’s superior performance, achieving a grafting efficiency of 700 plants/hour, a grafting success rate of 98.4%, and a grafted seedling survival rate of 96.8%. Its efficiency reaches 2.3 times that of manual labor, reducing labor costs by approximately 800 yuan per hectare. Compared with traditional vibrating-screen clip-feeding mechanisms, the cutting-clip feeding method employed in this study offers notable advantages in noise reduction, space efficiency, and cost savings. Its overall performance is outstanding, demonstrating exceptional practicality and economic value.

## Supporting information

S1 File Grafting machine SolidWorks 3D model. (PDF)

S2 Table Raw stress-strain curve data from polyethylene (PE) material tensile tests. (XLSX)

S3 Video Simulated video of cutting operations. (MP4)

S4 File Complete experimental design data and analysis for Box-Behnken response surface methodology. (XML)

S5 Table Test records for different cutting angles. (XLSX)
