# Supplementary figures and images for: Design and optimization of a butterfly-shaped grafting clip and cutting mechanism based on finite element simulation
Source: PLoS One. 2026 Jan 30;21(1):e0339854. doi: 10.1371/journal.pone.0339854 (PMC12858020; doi:10.1371/journal.pone.0339854)

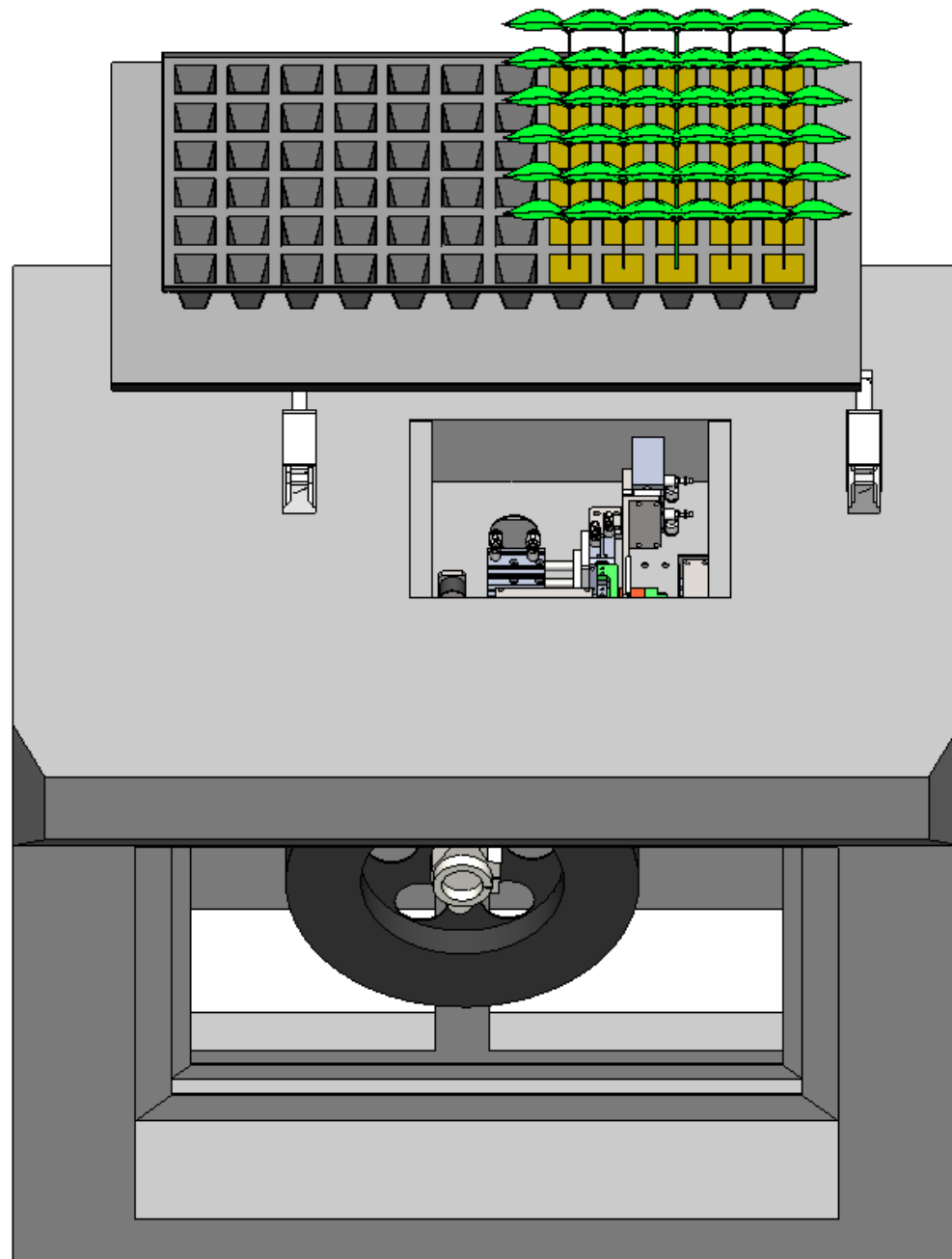

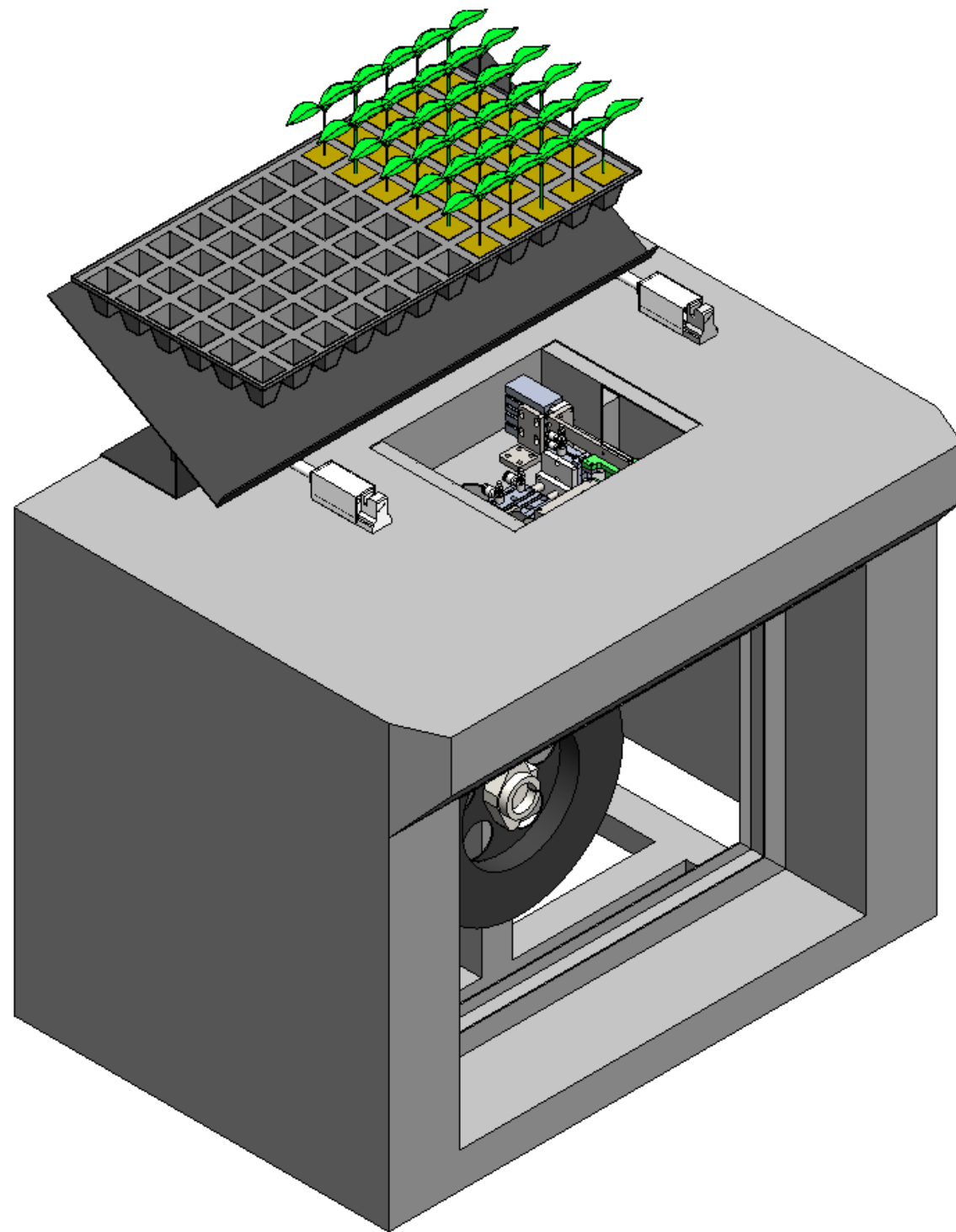

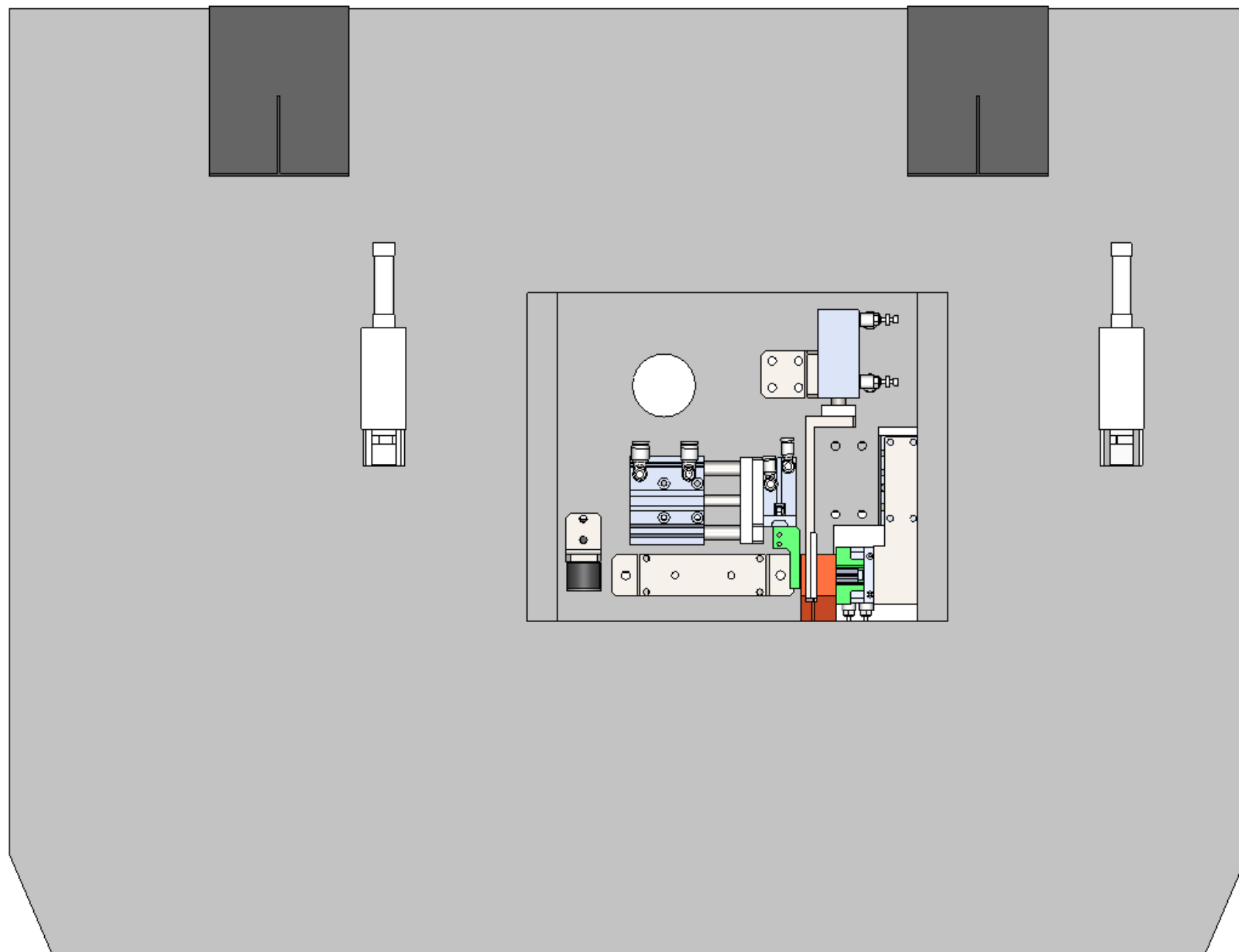

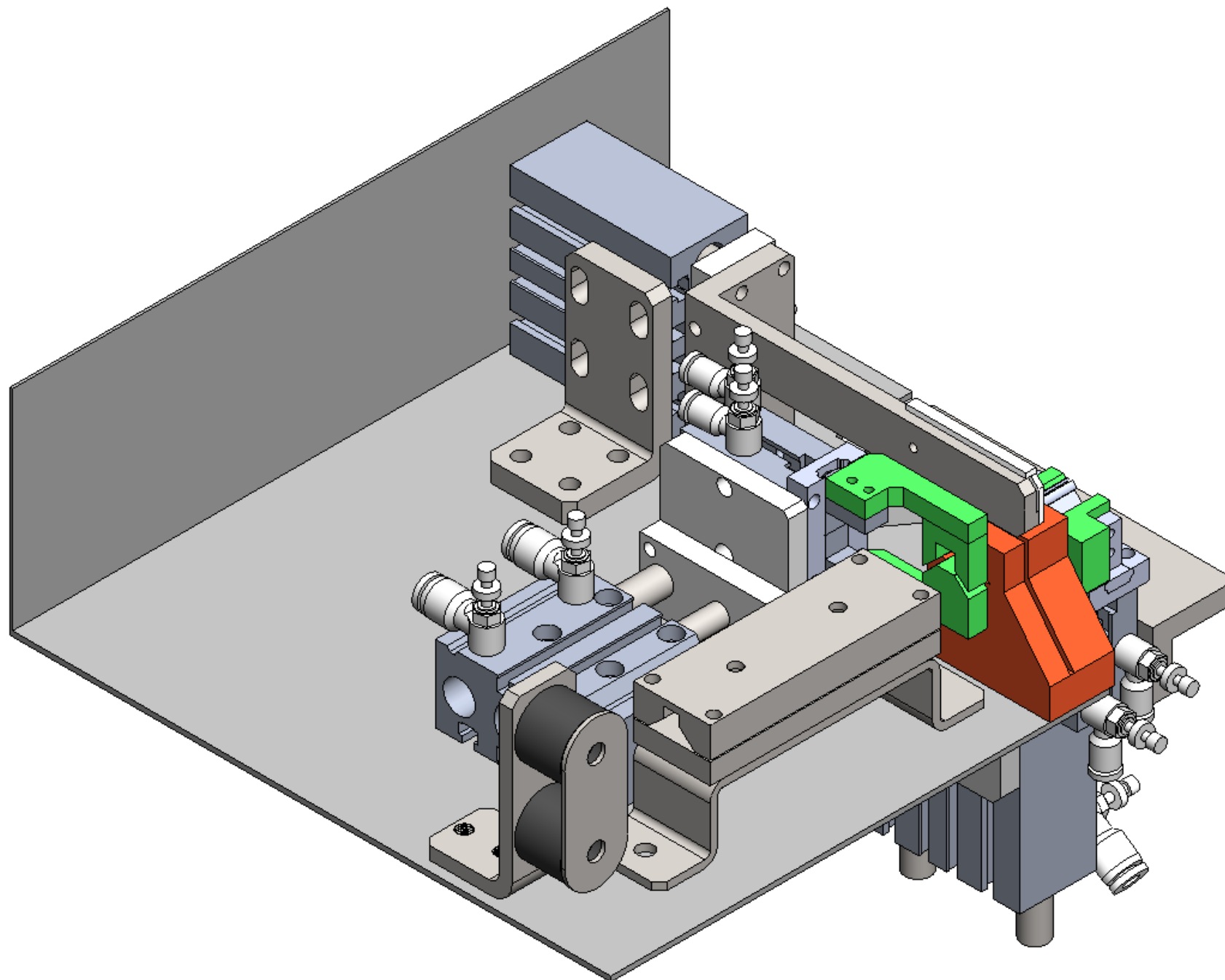

Supplement: S1 File — (PDF) [file pone.0339854.s001.pdf]
